# Perioperative outcomes of multiport or single-port, transperitoneal or retroperitoneal robot assisted radical nephroureterectomy: a narrative review

**DOI:** 10.3389/fonc.2025.1655703

**Published:** 2025-10-06

**Authors:** Pierre-Etienne Gabriel, Shahrokh F. Shariat, Morgan Rouprêt, John P. Sfakianos, Evanguelos Xylinas

**Affiliations:** ^1^ Department of Urology, Bichat Claude-Bernard Hospital, Assistance Publique -Hôpitaux de Paris Nord, University Paris Cité, Paris, France; ^2^ Department of Urology, Comprehensive Cancer Center, Medical University of Vienna, Vienna, Austria; ^3^ Sorbonne University, groupe recherche clinique (GRC) 5 Predictive Onco-Urology, Assistance publique des hôpitaux de Paris (AP-HP), Urology, Pitié-Salpêtrière Hospital, Paris, France; ^4^ Department of Urology, Icahn School of Medicine at Mount Sinai, New York, NY, United States

**Keywords:** robot assisted radical nephroureterectomy, multiport, single-port, transperitoneal, retroperitoneal

## Abstract

**Background:**

Robot-assisted radical nephroureterectomy (RARNU), performed via either a multiport or single-port approach through transperitoneal or retroperitoneal routes, is an increasingly utilized surgical method for patients with upper tract urothelial carcinoma.

**Materials and methods:**

A collaborative review of the literature available on Medline was conducted to report the perioperative outcomes of multiport or single-port, transperitoneal or retroperitoneal RARNU. A total of 31 references published between 2006 and 2023 were included.

**Results:**

The multiport transperitoneal robotic approach has been documented in 23 studies including between 10 and 3774 RARNU. Operative times ranged from 157 to 326 minutes, intraoperative complication rates from 0% to 7.3%, estimated blood loss from 68.9 mL to 380 mL and blood transfusion rates from 1.4% to 22.7%. Overall postoperative complication ranged from 11.9% to 43.8%, with major complications occurring in 0% to 15.1% of cases. Additionally, the length of hospital stay ranged from 2.3 to 10.3 days. The single-port transperitoneal robotic approach has been documented in 3 studies including between 1 and 12 RANU. Operative time ranged from 160 to 240 minutes, with 17% of patients requiring transfusions. The length of stay varied between 3 and 7 days. Finally, five retrospective studies, including between 2 and 12 patients treated with multiport retroperitoneal RARNU and between 2 and 20 patients with single-port retroperitoneal RARNU were reported, also with satisfactory results.

**Conclusion:**

Although prospective comparative studies are needed to confirm these results, RARNU approach, whether single-port or multi-port, transperitoneal or retroperitoneal, appears promising and safe.

## Introduction

Upper tract urothelial carcinoma (UTUC) represents 5-10% of all urothelial cancers (1). Although kidney-sparing surgery emerged for selected patients with low-risk UTUC, radical nephroureterectomy (RNU) with bladder cuff excision (BCE) remains the standard treatment for those with high-risk disease (2). Historically, the open approach has been considered as the standard for RNU due to the technical challenges in accessing the kidney, ureter, and bladder, but it was associated with significant postoperative morbidity (3). Thus, laparoscopic RNU was introduced in 1991 to enhance perioperative outcomes (4). Specifically, the use of laparoscopic RNU has been shown to decrease the risk of postoperative complication and the length of stay as compared to open procedures (5), with similar oncological outcomes in several retrospective studies (6, 7). However, the complexity of laparoscopic instrumentation and the steep learning curve associated with laparoscopic BCE have prevented this technique from being widely accepted by the urological community (8).

More recently, with the increasing adoption of robotic surgery in urological cancer (9), the use of the robotic approach has been proposed to facilitate distal ureter management (10). Since, multiport or single-port, transperitoneal or retroperitoneal robot-assisted radical nephroureterectomy (RARNU) emerged as the new standard of care for patients with high-risk UTUC (11, 12). Against this backdrop, we aimed to report the latest available evidence, with a focus on perioperative outcomes, of multiport or single-port RARNU using either a transperitoneal or retroperitoneal approach for patients with UTUC.

## Materials and methods

This collaborative narrative review was conducted using two separate online search engines, PubMed and Google Scholar, to identify relevant literature regarding RARNU in UTUC patients. In our research, we employed the following keywords either individually or in combination “robotic”; “radical nephroureterectomy”; “multiport”; “single-port”; “transperitoneal”; “retroperitoneal”. Articles were assessed for their relevance and methodology without any time restriction by three authors (PEG, JPS and EX). We focused exclusively on the perioperative outcomes of these four techniques, excluding oncologic results. RARNUs, with or without bladder cuff excision, were included in our review. This research excluded non-English literature, animal studies and correspondence/letters. Given the lack of available data, small series were also included in this review. In total, 31 references published between 2006 and 2023 were included in our result section.

## Results

### Transperitoneal RARNU

#### Multiport transperitoneal RARNU

The perioperative outcomes of the multiport transperitoneal RARNU approach have been documented in 23 studies, including between 10 to 3,774 patients (10, 13–34); **Table 1**. Regarding operative time, 18 studies (10, 13–17, 23–34) reported a mean/median duration between 157 and 326 minutes. Intraoperative complications, documented in 7 studies (10, 13, 14, 16, 18, 32, 34), ranged from 0% to 7.3%. Estimated blood loss was reported to be between 68.9 mL and 380 mL across 17 studies (10, 13–17, 24–34). Blood transfusion rates varied from 1.4% to 22.7% in 8 studies (10, 13, 15, 16, 21, 28, 31, 33). Overall postoperative complication rates ranged from 11.9% to 43.8% (10, 13–18, 21, 23, 28–30, 32–34), while major complications were reported between 0% and 15.1% (10, 13–17, 20, 23, 28, 29, 32–34). Additionally, length of hospital stay was reported in 21 studies, with durations ranging from 2.3 to 10.3 days (10, 13–22, 24, 26–34).

**Table 1:** Perioperative results of transperitoneal multiport robot assisted radical nephroureterectomy.

Studies	RARNU (n)	Operative time (median, min)	Intraoperative complications (%)	Estimated blood loss (median, mL)	Blood Transfusions (%)	Conversion (%)	Overall complications (%)	Major complications (%)	Length of stay (median, days)	Perioperative mortality (%)	Positive surgical margins (%)
Zargar, 2014 (10)	31	300*	0	200	0	0	22.5	3.2	5	NR	3.2
Ambani, 2014 (13)	22	298*	5	380*	9.1	0	32	5	3.1*	NR	9
Lee, 2019 (14)	124	248.5*	7.3	200*	NR	NR	13.7	0.8	10.3*	NR	NR
Melquist, 2016 (15)	37	306	NR	150	8	5	25	10	5	NR	0
Gabriel, 2023 (16)	70**	157	5.7	200	1.4% (intraoperative) 5.7% (postoperative)	0	12.9	7.1	4	NR	8.6
Grossmann, 2023 (17)	473	240	NR	100	NR	0	22	5.1	4	NR	3
Pearce, 2016 (18)	2286	NR	2	NR	NR	NR	19	NR	4	NR	NR
Kenigsberg, 2021 (19)	1129	NR	NR	NR	NR	3.3	NR	NR	3	0.6	11.1
Li, 2021 (20)	141	NR	NR	NR	NR	NR	NR	2.8	7.73*	NR	NR
Trudeau, 2014 (21)	715	NR	NR	NR	13	NR	11.9	NR	5.6	NR	NR
Clements, 2018 (22)	315	NR	NR	NR	NR	NR	NR	NR	4.2*	NR	NR
Tinay, 2016 (23)	3774	286	NR	NR	NR	NR	41.5	8.6	NR	NR	NR
Hu, 2015 (24)	18	255.17*	NR	68.9*	NR	NR	NR	NR	6.79*	NR	NR
Ting, 2021 (25)	10	240*	NR	120*	NR	NR	NR	NR	NR	NR	0
Ye, 2020 (26)	29	300	NR	100	NR	0	NR	NR	4.86*	NR	6.9
Hemal, 2011 (27)	15	183.9*	NR	103*	NR	0	NR	NR	2.7*	NR	0
Lim, 2013 (28)	32***	250.1*	NR	263*	6.3	0	28.1	6.2	6.2*	NR	3.1
Patel, 2018 (29)	87	184.4-232.1*	NR	122.6- 156.6*	NR	NR	13.3-17.5	3.3- 3.5	2.3-2.6*	NR	21.04-11.94
Pugh, 2013 (30)	43	247	NR	131*	NR	NR	14	NR	3	NR	2
Eandi, 2010 (31)	11	326	NR	200	9.1	0	NR	NR	4.7	NR	0
Park, 2009 (32)	11	193-247.3*	0	106.7- 270*	NR	0	0	0	7-8.4*	NR	0
Zeuschner, 2021 (33)	66	188	NR	150	22.7	4.5	40.9	15.1	9*	NR	1.5
Campi, 2019 (34)	66	195	3	200	NR	0	43.8	6	5	NR	6

*mean.

**3 retroperitoneal approach.

***13 laparoscopic single site.

NR, not reported in the study.

#### Single-port transperitoneal RARNU

The single-port transperitoneal robotic approach has been documented in 3 studies including between 1 and 12 RANU (**Table 2**). Lee et al. reported satisfactory perioperative outcomes in a retrospective cohort of 68 patients who underwent robot-assisted surgery using a single-port technique, including 12 RARNU procedures. The mean operative time was 227 minutes, with an estimated blood loss of 248 mL, a perioperative transfusion rate of 17%, and a mean hospital stay of 4 days (35). Additionally, Kim et al. share their experience with single-port robotic surgery performed by a single surgeon in a second retrospective study. Among 120 urological procedures, 5 patients underwent transperitoneal single-port RARNU. In this study, the operative time ranged from 160 to 240 minutes, blood loss between 100 and 200 mL, and hospital stay varied from 3 to 7 days (36). Finally, Abaza et al. describe, in a third retrospective study from 2021, their first 100 single-port robotic surgeries, including 59 prostatectomies, 18 partial nephrectomies, 12 pyeloplasties, 4 nephrectomies, 4 adrenalectomies, 2 partial cystectomies, and 1 transperitoneal RNU. Although specific perioperative outcomes for UTUC are not detailed, the overall results were promising, highlighting the feasibility and potential benefits of a single-port approach (37).

**Table 2:** Perioperative results of transperitoneal single-port robot assisted radical nephroureterectomy.

Studies	RARNU (n)	Operative time (median, min)	Intraoperative complications (%)	Estimated blood loss (median, mL)	Blood Transfusions (%)	Conversion (%)	Overall complications (%)	Major complications (%)	Length of stay (median, days)	Perioperative mortality (%)	Positive surgical margins (%)
Lee, 2011 (35)	12	227*	NR	248*	17	0	NR	NR	4*	NR	NR
Kim, 2023 (36)	5	160-240	NR	100-200	NR	0	NR	NR	3-7	NR	NR
Abaza, 2021 (37)	1	NR	NR	NR	NR	NR	NR	NR	NR	NR	NR

*mean.

NR, not reported in the study.

### Retroperitoneal RARNU

While the transperitoneal approach remains the traditional route (3), there is increasing interest in the retroperitoneal approach for RARNU due to its potential benefits. Thus, Sparwasser et al. conducted in 2023 the first direct comparison between retroperitoneal and transperitoneal RARNU. The analysis included perioperative patient data from 24 transperitoneal RNU and 12 retroperitoneal RNU cases. While intraoperative (16.4% vs 0%, p = 0.35) and postoperative (25% vs 12.5%, p = 0.64) complications showed no significant differences, retroperitoneal route was significantly associated with reduced surgery time (p < 0.05) and shorter length of stay (p < 0.05) compared to transperitoneal RNU (38). Regarding the surgical technique, retroperitoneal RNU has been described using both multiport and single-port robotic techniques.

#### Multiport retroperitoneal RARNU

To date, there have been very few reported cases of complete RARNU with BCE using the multiport retroperitoneal approach (**Table 3**). In 2006, Rose et al. reported in a retrospective study the first experience including 2 patients treated by robotic retroperitoneoscopic RNU using the Da Vinci Surgical System. Both procedures were successfully completed with the robot without conversion. Mean operative time was 182.5 min and estimated blood loss was 75 ml. In this study, postoperative recovery was uneventful (39). More recently, a retrospective analysis reported five patients who underwent RARNU with BCE exclusively within the retroperitoneal space. In this study, UTUC was localized to the distal ureter in two cases and to the kidney in three. None of the patients with UTUC had positive surgical margins. Regarding perioperative outcomes, no intraoperative adverse events of grade ≥2 were reported, and the median estimated blood loss was 150 ml. Additionally, no patients experienced postoperative complications classified as Clavien–Dindo grade ≥ 3a. The median hospital stay was 5.4 days, with no readmissions within 30 days. It is worth mentioning that the authors noted that intraoperative redocking was required for managing the distal ureter, which took 7 minutes (40). Finally, Sparwasser et al. reported a series of 12 RARNU. In the study, the mean operative time was 192 minutes, with a perioperative transfusion rate of 8.3%, and a mean hospital stay of 5.75 days (38).

**Table 3:** Perioperative results of retroperitoneal multiport robot assisted radical nephroureterectomy.

Studies	RARNU (n)	Operative time (median, min)	Intraoperative complications (%)	Estimated blood loss (median, mL)	Blood Transfusions (%)	Conversion (%)	Overall complications (%)	Major complications (%)	Length of stay (median, days)	Perioperative mortality (%)	Positive surgical margins (%)
Rose, 2006 (39)	2	182.5*	0	75*	NR	0	NR	NR	5.6*	0	0
Sparwasser, 2022 (40)	5	189.2*	20	150	20	0	NR	0	5.4*	0	0
Sparwasser, 2023 (38)	12	192*	0	NR	8.3	0	NR	25	5.75*	0	0

*mean.

NR, not reported in the study.

#### Single-port retroperitoneal RARNU

Currently, there is also limited literature on RNU with BCE using the single-port robotic system, and the available studies involve a small number of patients (**Table 4**). Thus, in 2023, Pellegrino et al. presented results for 18 patients treated using a supine anterior retroperitoneal access technique with the da Vinci Single-Port robotic platform, which included 2 RNU with BCE. The findings suggested that this approach was feasible and safe, with low complication rates, reduced postoperative pain, and earlier discharge (41). Additionally, Bang et al. reported perioperative outcomes in 20 patients who underwent single-port RARNU with BCE. In this retrospective study, the median console time was 106 minutes and 40 seconds, with expected blood loss of 122.50 ± 75.18 mL. Postoperative outcomes were also satisfactory, as none of these 20 patients experienced complications according to the Clavien-Dindo scale (42).

**Table 4:** Perioperative results of retroperitoneal single-port robot assisted radical nephroureterectomy.

Studies	RARNU (n)	Operative time (median, min)	Intraoperative complications (%)	Estimated blood loss (median, mL)	Blood Transfusions (%)	Conversion (%)	Overall complications (%)	Major complications (%)	Length of stay (median, days)	Perioperative mortality (%)	Positive surgical margins (%)
Pellegrino, 2023 (41)	2	NR	NR	NR	NR	0	NR	NR	NR	NR	0
Bang, 2023 (42)	20	150.5*	0	122.5*	0	0	0	0	4.5	0	NR

*mean.

NR, not reported in the study.

## Discussion

Given the widespread adoption of robotic in the surgical management of urological cancers, we compiled in this narrative review the perioperative results of multiport transperitoneal RARNU. We also incorporated data on single-port transperitoneal procedures and retroperitoneal techniques (both multiport and single-port). Overall, our analysis shows that the multiport transperitoneal approach is well documented and associated with satisfactory perioperative outcomes, supporting its large-scale adoption compared to laparoscopic techniques. These findings are further supported by comparative analyses. The perioperative outcomes of multiport transperitoneal RARNU have been compared to those of laparoscopic RNU in a recent meta-analysis and systematic review. Thus, O’Sullivan et al., reported in a meta-analysis including 10 studies and employing the fixed-effects model of Mantel-Haenszel no significant difference between the two techniques in terms of overall postoperative morbidity, including major complications, operative time, estimated blood loss, intraoperative complications, and postoperative surgical margins. However, a slight reduction in length of stay and perioperative mortality was observed in favor of the RARNU group (43). Additionally, a systematic review including a total of 8,470 patients who underwent RARNU and 19,872 patients who underwent laparoscopic RNU analyzed data from 12 comparative original studies. Although the robotic procedure was linked to a longer operative time, the results indicated that it was associated with fewer overall complications and a shorter hospital stay compared to laparoscopic RNU (44).

With regards to other robotic techniques, the currently available data consist only of a few small retrospective series. Compared to transperitoneal multiport RARNU, the transperitoneal single-port route has also demonstrated satisfactory perioperative outcomes, with the potential advantage of reducing postoperative pain due to the single incision and facilitating early discharge. The retroperitoneal approach, whether multiport or single-port, may be a preferable option for patients with a history of abdominal surgery to avoid intraperitoneal adhesions (45). However, the difficulty of this access route lies in working within a confined space, which significantly limits the triangulation of multiport instruments and dexterity (46). Moreover, a major hurdle to this approach is the challenge of instrument placement and the potential conflicts that may arise, making the procedure complex and difficult to replicate (41). In any case, the optimal approach needs to be adapted to each patient, and the choice must take into account the medical history, the tumor characteristics, and the surgical expertise.

In addition to perioperative benefits, the robotic approach offers several advantages for both the surgeon and the procedure, including enhanced ergonomics, greater precision, elimination of tremors, the ability to operate in 3D vision, and increased degrees of freedom in instrument mobility, enabling fine and accurate movements (47). These features may contribute to better oncological outcomes and account for the satisfactory perioperative results reported in this review. With regards to the surgical procedure, the robotic approach simplifies endoscopic excision of the bladder cuff, whereas laparoscopic procedures often require an iliac incision. In fact, a multicenter study involving data from 17 centers and 276 patients (185 robotic, 91 laparoscopic) demonstrated that patients undergoing RARNU were significantly more likely to have BCE endoscopically (81% vs. 63.7%, P = 0.003), providing strong evidence in favor of promoting robotic surgery for the management of RNU (48).

Regarding oncological outcomes, several previous studies investigated the impact of the surgical RNU approaches on survival outcomes and found similar progression-free survival (PFS), cancer-specific survival (CSS), and overall survival (OS) among the three approaches (open, laparoscopic and multiport transperitoneal robotic), despite variations in statistical methods, cohort sizes, and study designs (14, 17, 22, 49). Additionally, the most recent meta-analysis by Vecchia et al., which investigated the effect of the surgical technique on PFS and CSS in approximately 87,000 patients, observed no significant difference (12). However, the primary concern with the robotic approach relates to the risk of intravesical recurrence. In fact, Grossmann et al. reported in a recent multicenter study, which involved 756 patients and used propensity score matching, that although Kaplan–Meier and log-rank analyses found similar RFS, CSS, and OS across transperitoneal multiport RARNU, LRNU, and ORNU groups, the intravesical RFS was significantly higher with open surgery. Additionally, using multivariable regression analyses, LRNU and RARNU were independently associated with worse intravesical RFS (HR 1.66, 95% CI 1.22–2.28, *p* = 0.001 and HR 1.73, 95%CI 1.22–2.47, *p* = 0.002, respectively) (17). Thus, the latest EAU guidelines advise caution, given the potentially higher risk of intravesical recurrence associated with both laparoscopic and robotic RNU compared to the open approach (2). However, further comparative studies are needed to confirm this precaution.

Finally, beyond perioperative and oncological outcomes, cost considerations remain another key factor influencing the widespread adoption of robotic surgery. Although the initial costs for acquiring and maintaining robotic systems are substantial, some indirect benefits—such as shorter hospital stays and fewer complications (43, 44)—may help offset these expenses. However, a recent study by Di Bello et al., including 1,138 RNU procedures, found that RARNU was significantly associated with higher hospital costs compared to open surgery ($64,761 vs. $54,768, p<0.001). Additionally, even after adjusting for multiple variables, RARNU remained an independent predictor of increased hospital costs (HR: 1.13; P<0.001), which likely represents the main obstacle to its large-scale implementation (50). However, the widespread adoption of robotic procedures across all surgical specialties, as well as the entry of new competitors and the standardization of consumables, are expected to further reduce the overall costs of these procedures.

As a narrative review, our report has certain limitations, including reliance on retrospective studies, heterogeneity in study designs and outcome measures, and small sample sizes in certain surgical approaches. Future research is needed to better evaluate and compare these four techniques, particularly through multicenter prospective studies assessing long-term oncologic outcomes, as well as cost-effectiveness analyses and the impact on patient quality of life associated with the use of these new approaches.

## Conclusion

This narrative review compiled the perioperative outcomes of RARNU performed via transperitoneal or retroperitoneal approaches, using either single-port or multi-port techniques in patients with UTUC. We found that the multi-port transperitoneal technique is well-documented, safe, and should be part of the surgical arsenal for high-risk UTUC patients. Regarding the single-port transperitoneal approach and the retroperitoneal approach with either single-port or multi-port techniques, the current number of available studies is limited. Further research involving a larger number of patients is needed to better assess these surgical options.
